# Effects of biochar combined with nitrification inhibitors on NH_3_ and N_2_O emission under different water conditions from vegetable soils

**DOI:** 10.3389/fmicb.2025.1547979

**Published:** 2025-03-17

**Authors:** Zhenyuan He, Haohao LÜ, Yuying Wang, Hangjie Yuan, Yuxue Liu, Neng Li, Lili He

**Affiliations:** ^1^State Key Laboratory for Managing Biotic and Chemical Threats to the Quality and Safety of Agro-products, Institute of Environment Resource Soil and Fertilizer, Zhejiang Academy of Agricultural Sciences, Hangzhou, China; ^2^Bamboo Home Engineering Technology Research Center of National Forestry and Grassland Administration, China National Bamboo Research Center, Hangzhou, China; ^3^School of Life Science, Henan University of Science and Technology, Xinxiang, China; ^4^Agricultural Technology Extension Center of Hangzhou, Hangzhou, China; ^5^Anhui Province Agricultural Waste Fertilizer Utilization and Cultivated Land Quality Improvement Engineering Research Center, Chuzu, China

**Keywords:** biochar, nitrification inhibitor, water contents, active nitrogen gases, *amoA*

## Abstract

Soil nitrogen loss through NH_3_ volatilization and N_2_O emissions is a crucial issue in soil ecosystems. In this study, we explored the effects of biochar and the nitrification inhibitor DMPP (dimethyl-phenyl-piperazinium, a nitrification inhibitor) in vegetable soils under 60 and 200% WHC (water holding capacity). Five treatments were set: CK (control), urea (N), urea + biochar (N + C), urea + nitrification inhibitor (N + DMPP), and urea + nitrification inhibitor + biochar (N + C + DMPP). Results found that biochar promoted soil nitrification and ammonia volatilization under both moisture conditions, with higher NH_3_ rate accumulation at 200% WHC. DMPP maintained high NH_4_^+^-N concentration and increased soil ammonia volatilization, but effectively reduced N_2_O emissions, especially at 200% WHC. The N + C + DMPP treatment further significantly decreased N_2_O cumulative emissions compared to N + DMPP. QPCR results showed that N + C treatment significantly increased AOB (ammonia-oxidizing bacteria) copies compared to N treatment. Applying DMPP alone or with biochar reduced AOB copies by 50.0 and 45.7%, respectively. Soil ammonia-oxidizing archaea (AOA) responded oppositely to DMPP; AOA amounts in N + DMPP and N + C + DMPP treatments increased significantly during the culture. At 60% WHC, the greenhouse effect potential of N + DMPP and N + C + DMPP treatments were 39.0 and 43.2% lower than N, respectively. At 200% WHC, their GWP were decreased by 13.8 and 0.08% compared to N. Adding biochar alone increased the soil’s greenhouse potential at both water contents. In conclusion, using nitrification inhibitors alone or in combination with biochar is more effective in reducing the greenhouse effect potential of soil active nitrogen emissions.

## 1 Introduction

Ensuring food security and addressing climate change and environmental degradation are the three major challenges facing countries around the world today. With the large-scale production and application of chemical nitrogen fertilizer, global food production has increased by nearly 150% in the past 50 years, and the average nitrogen (N) fertilizer use has reached 180 kg hm^−2^, 75% higher than the world average (Tilman et al., 2002). At the same time, a large amount of greenhouse gases and reactive nitrogen loss emitted during field food production have promoted the process of global climate warming and environmental deterioration. N_2_O emissions are among the most serious environmental consequences of nitrogen fertilizer loss (Wang et al., 2024). Although it exists in trace amounts, its contribution rate to the greenhouse effect reaches 5%, and it has a strong warming potential, which is one of the important greenhouse gases leading to global warming. Studies have shown that about 25–40% of the urea N applied to soil is converted into NH_3_ and volatilized into the atmosphere (Bi et al., 2023). NH_3_ will lead to the acidification of soil and surface water and is the main contributor of haze or gray haze in the air. The control of atmospheric active nitrogen pollutants is an inevitable choice to solve the sustainable development of China’s agricultural system, improve the utilization rate of nitrogen fertilizer, and realize the win-win economic and ecological benefits of farmland fertilization.

As a kind of solid material with high carbon content, biochar will interfere with soil N behavior due to its unique properties and interaction with soil components after application (Lehmann et al., 2021; Liu et al., 2024). Due to its well-developed pore structure, large specific surface area, cation exchange capacity and other characteristics, biochar can absorb NH_3_ after input into the soil, and enhance the retention of ammonium ions (NH_4_^+^) by improving the cation substitution capacity of the soil, and also improve the nitrification rate of the soil, thus affecting the N loss and N_2_O emission of the soil (Zhang et al., 2025). Some scholars have found that when the biochar application rate exceeds 10 t ha^−1^, the cost of ecological services like N_2_O emission reduction, nitrate leaching reduction, and carbon sequestration is lower than the input cost of biochar (Smith et al., 2019). In order to achieve the best agronomy, environment and economic benefits, it is necessary to prepare lower-cost biochar or to apply high-efficiency biochar mixtures, such as coupled with N fertilizer synergists. Nitrification inhibitors (NIs), such as 2-chloro-6-trichloromethyl pyridine (nitrapyrin, NP), dicyandiamide (DCD), and 3, 4-dimethylpyrazole phosphate (DMPP), are cost-effective N fertilizer synergists that can inhibit the nitrogen nitrification process (Liu et al., 2025). NI can inhibit the emission of N_2_O and NO into the atmosphere by nitrification to varying degrees (Bachtsevani et al., 2021). Reports found that there is a strong correlation between NH_3_ and N_2_O emission in farmland soil (Martins et al., 2017), and Ma et al. (2020) also found that the total amount of NH_3_ volatilization in soil was significantly negatively correlated with the total amount of N_2_O emission (*r* = −0.859, *p* < 0.01). Since NI inhibits nitrification by extending the retention time of ammonium N in soil to varying degrees, it may increase the risk of NH_3_ volatilization emission. Biochar has a large specific surface area, has a good adsorption effect on ammonium N, but it is difficult to inhibit the negatively charged ions NO_2_^−^ and NO_3_^−^ (Lawrinenko and Laird, 2015). Whether the combined application of the two can play a positive interaction and the related mechanism need to be studied.

Soil water status is an extremely important factor affecting N_2_O emission, and it can regulate the effective utilization rate of O_2_, thus affecting the nitrification and denitrification processes in soil (Liu et al., 2022). It was reported that the N_2_O emission of soil decreased significantly under 100% WFPS and 125% WFPS (Wang et al., 2023). Soil moisture state also affects ammonia volatilization loss. Yang et al. (2019) found that the increase of soil water content may increase ammonia volatilization loss through accelerating urea hydrolysis and promoting microbial metabolism. However, the effects of biochar combined with nitrification inhibitors on soil N_2_O emission and ammonia volatilization loss under different water content and the mechanism of interaction with related microorganisms are still lacking. The objective of this study was to clarify the specific effects of biochar and DMPP alone and in combination on NH_3_ volatilization and N_2_O emissions in soil, and determine whether the use of biochar and DMPP can help reduce greenhouse gas emissions and mitigate the greenhouse effect under different moisture conditions and probe into the relevant microbiological mechanisms through indoor control simulation experiment (constant temperature and light conditions).

## 2 Materials and methods

### 2.1 Materials

The soil used for testing was collected from Xucun Town, Haining City, Zhejiang Province (30°31′N, 120°19′E). The topsoil (0–20 cm) was collected in April 2021, and residual gravel, crop roots, and debris in the fresh soil were removed. After natural air drying, the soil was screened using 10 meshes. The raw material of biochar is made from rice straw, which is pyrolyzed with a heating rate of 5°C min^−1^ and final temperature of 500°C in carbonization furnace for 3 h. The basic physical and chemical properties are as follows: total carbon 610 g kg^−1^, total nitrogen 14.3 g kg^−1^, soil cation exchange capacity (CEC) 18.9 cmol kg^−1^, pH 9.20. The nitrogen fertilizer was urea, which was produced by Sinopharm Group Co., Ltd., with 46% nitrogen content. Nitrification inhibitor 3, 4-dimethylpyrazole phosphate (DMPP) was selected for analytical purity and was produced by Macklin Biotechnology Company.

### 2.2 Methods

The soil NH_3_ and N_2_O emissions were studied using aerobic incubation experiments. Biochar, DMPP, or a combination of biochar and DMPP was added to urea, and two water conditions were set: 60% WHC (maximum field water capacity) and 200% WHC. Five treatments for each water condition: CK (control), urea (N), urea + biochar (N + C), urea + nitrification inhibitor (N + DMPP), urea + nitrification inhibitor + biochar (N + C + DMPP), each treatment was set up with three replicates, where the urea addition amount was 0.3 g N kg^−1^, and the biochar addition amount was 2% of the soil mass; biochar was passed through a 1 mm sieve prior to the incubation experiment. The amount of DMPP added was recognized as the optimal dosage of 1% of the amount of urea nitrogen. Fresh soil equivalent to 50-g air-dried soil and the corresponding biochar rate were placed in 500 mL conical flasks and shaked thoroughly to ensure the mixtures uniform and then incubated at 30°C in the dark for 42 days after 7-day pre-incubation (Duan et al., 2017). The flasks were covered with a polyethylene film and then punctured with small holes to maintain aerobic conditions. Deionized water was added every 2–3 days to compensate for water loss at least 1 day before sampling to avoid gas emission.

### 2.3 Collection and determination of N_2_O emission, NH_3_ emission and soil DNA

N_2_O gas was collected at 1, 2, 5, 7, 9, 11, 14, 17, 21, 23, 27, 29, 37, and 42-day intervals using a gas-tight syringe. Prior to gas sampling, the head-space air within the flasks was meticulously purged with ambient air for 15 min at a flow rate of 200 mL per minute. Immediately afterward, the flasks were sealed with silicone rubber stoppers (NQ-704 silicone adhesive sealant) equipped with butyl rubber septa and then incubated for 5 h. For each measurement, gas samples were extracted from the flasks via a three-way stopcock using a 25-mL air-tight syringe. Following sample withdrawal, the flasks were purged with ambient air and left open (He et al., 2016). The concentration of the N_2_O was immediately determined by a gas chromatograph (Agilent 7890) and detected by ECD. The carrier gas employed was a 5% argon-methane mixture, flowing at a rate of 40 mL per minute, and the column temperature was maintained at 40°C. Compressed air was utilized as the standard gas, with a designated value of 313 ppbv. The concentrations of N_2_O were determined by comparing the peak areas of the samples with those of the reference gases supplied by Hangzhou Jingong Special Gas Factory. (calculated by Equations (1) and (2)).

NH_3_ gas was collected at 1, 2, 3, 4, 6, 8, 10, and 14-day intervals. The NH_3_ flux was measured by a Dräger flow-through chamber technique (Kelsch et al., 2024). Before gas sampling, the flasks were removed from the incubator and uncovered to conduct gas exchange between the inside and outside of the incubation flasks for 30 min (the NH_3_ concentration in the headspace reached equilibrium with the ambient air during this period via pre-incubation). The flasks were then sealed with silicone rubber stoppers with one inlet hole equipped with an air check valve to avoid pressure changes, which could affect the NH_3_ flux measurements, and an outlet hole equipped with a three-way valve connected to a Dräger tube. The flasks were then immediately capped and incubated for 24 h. The NH_3_ concentration of each sample was measured using a Dräger-Tube Sensor (Drägerwerk AG, Lübeck, Germany), which contained a solid-phase acid compound and bromophenol blue pH indicator and was calibrated by the manufacturer. A Dräger tube was inserted into the corresponding Dräger Gas Detector hand pump (semicontinuous suction characteristics). Ambient air was sucked into 100-mL through flasks using a hand pump. The NH_3_ concentration was immediately displayed as the total length of the color change. (calculated by Equations (3) and (4)).

Subsamples were destructively sampled at 0, 7, 14, 21, 28, 35, and 42-day intervals, respectively, which were treated with 2 mol L^−1^ KCl solution to extract NH_4_^+^ and NO_3_^−^ and measure soil pH.

The subsamples were destructively sampled at the 1, 7, and 42-day intervals for and DNA extraction which were immediately stored at-20°C. Total soil genomic DNA was extracted from each soil sample using a PowerSoil DNA isolation kit (Mo Bio Laboratories, Carlsbad, CA, United States), following the manufacturer’s instructions. Subsequently, the quantification of functional genes (AOA, AOB, and Urec) was performed by real-time quantitative PCR to obtain a view of the specific enzymes for NH_4_^+^/NH_3_ production and emission (Applied Biosystems, Foster City, CA). A standard curve was constructed using plasmid DNA from one representative clone containing each target gene.

### 2.4 Determination items and methods

1.  The calculation formula for calculating the soil N_2_O emission rate was as follows:

(1) $$F=\rho \times \Delta ∁/\Delta t\times 273/\left(273+T\right)\times V/m$$

where *F* is the N_2_O emission rate (μg N kg^−1^ h^−1^); Δ*C/*Δ*t* is the rate of N_2_O concentration in the culture device (10^9^ N_2_O-N h^−1^); *V* is the volume of the culture flask (m^3^); *T* stands for culture temperature (°C); m is the dry base weight (kg) of the cultured soil.

Cumulative N_2_O emissions from soil are calculated by the following formula:


(2) $$E=\overset{i}{\underset{i+1}{\sum }}\left(f_{i}+f_{i+1}\right)/2\times \left(t_{i}+t_{i+1}\right)$$

where *E* is the cumulative emission of N_2_O (μg N kg^−1^); $f_{i}$ and $f_{i+1}$ are the rate of N_2_O emission during culture time $t_{i}$ and $t_{i+1}$ .

2.  The calculation formula for calculating the ammonia volatilization rate is as follows:

(3) $$F_{N}=C\times \frac{M}{V_{m}}\times \frac{273}{273+T}\times \frac{V}{m}\times \frac{1}{t}\times k$$

where FN is the ammonia volatilization rate (mg N kg^−1^ day^−1^); C is the concentration determined by ammonia detection tube (mg L^−1^); M is the molar mass of N (g moL^−1^); *V*_m_ is the molar volume of the gas, which is 22.4 L moL^−1^ under standard conditions; *T* is the culture temperature (°C); *V* is the volume of gas in the culture device (mL); *m* is soil mass (g); *t* is time (day); *k* is the conversion factor, which is the ratio of unit mass to unit volume.


(4) $$E_{N}\left(\mathrm{mg}\;N\;{\mathrm{kg}}^{-1}\right)=\int_{i-1}^{i}{F_{NH}}_{3}$$

Where *E*_N_ indicates the cumulative amount of ammonia volatilization (mg N kg^−1^); *i* represents the days of culture (day); *F*_NH3_ stands for ammonia volatilization rate (mg N kg^−1^ day^−1^).

3.  Potential greenhouse effect of active soil nitrogen emissions.

(5) $$\begin{array}{l} \left(GWP\right)=\mathrm{direct}\;GWP\;\left({CO}_{2}-\mathrm{eq}\;\mathrm{mg}\;{\mathrm{kg}}^{-1}\right)+\\ \mathrm{indirect}\;GWP\;\left({CO}_{2}-\mathrm{eq}\;\mathrm{mg}\;{\mathrm{kg}}^{-1}\right) \end{array}$$

Among them, the indirect N_2_O emission is calculated as follows: indirect N_2_O = EF × NH_3_-N, EF is the N_2_O emission coefficient of NH_3_ volatilization loss, and its value is 0.01 mg N_2_O-N mg^−1^ N. According to the IPCC (2013) report, approximately 1% (0.2%–5%) of the emitted NH_3_-N is deposited on land and converted into N_2_O, which is used to estimate the GWP. Its calculation formula is as follows (calculated by Equations (5) and (6)):


(6) $$GWP\;\left({CO}_{2}-\mathrm{eq}\;\mathrm{mg}\;{\mathrm{kg}}^{-1}\right)=298\times N_{2}O\;\left(\mathrm{mg}\;N\;{\mathrm{kg}}^{-1}\right)÷28\times 44$$

where N_2_O-N (mg kg^−1^) is the cumulative emission of N_2_O-N during the culture period.

### 2.5 Data analysis and processing

OriginPro 2019 mapping software and IBM SPASS 22.0, were used to carry out univariate analysis of soil NH_3_ cumulative emissions, N_2_O emissions, and soil physicochemical properties in each fertilization period between the treatments. One-way analysis of variance (ANOVA) was used to check for quantitative differences between treatments. Different cases and English letters indicate significant differences among the treatments (*p* < 0.05).

## 3 Results and discussion

### 3.1 Effects of biochar and DMPP on inorganic nitrogen conversion under different water conditions

#### 3.1.1 NH_4_^+^-N content

**Figure 1:**
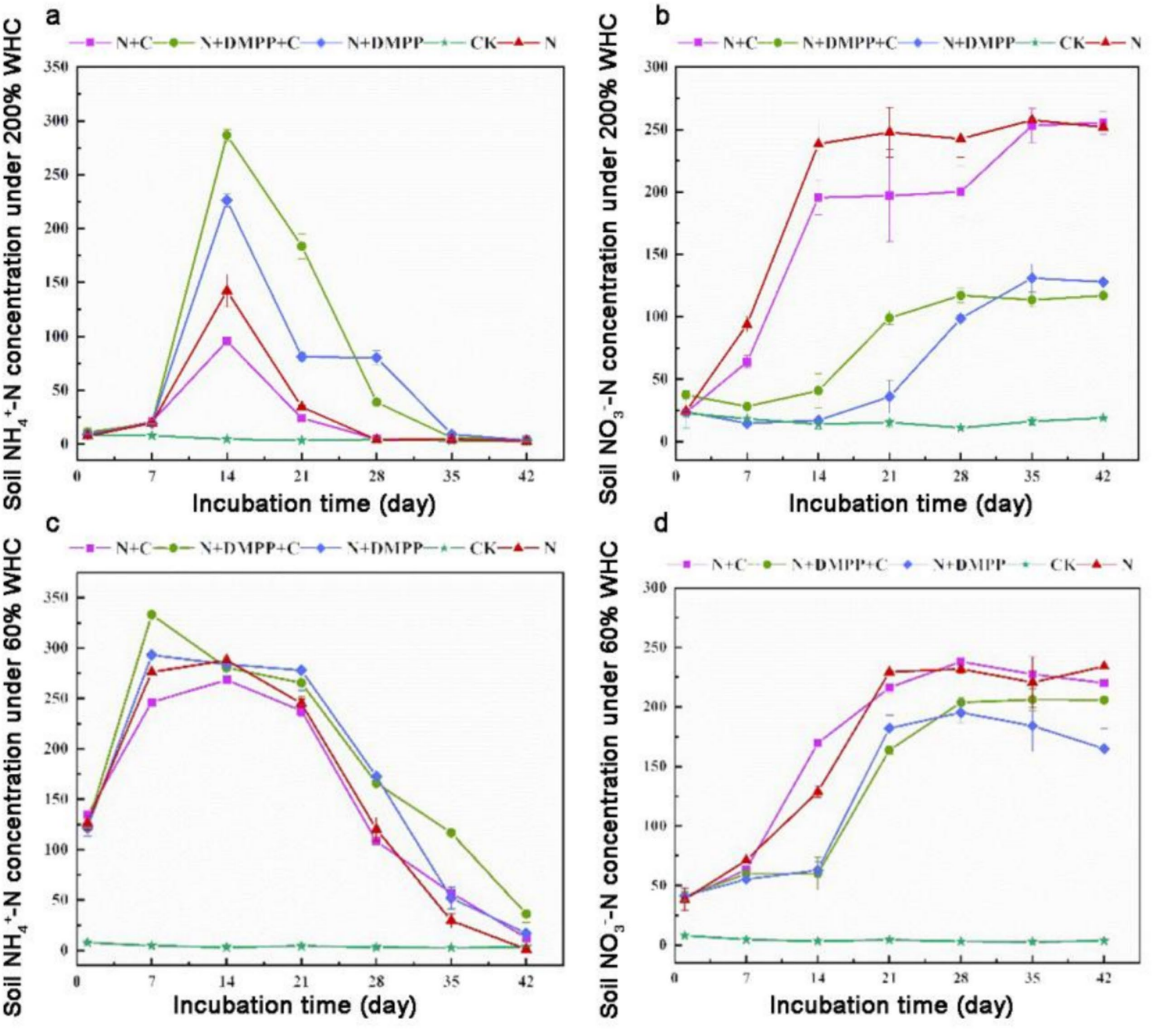
Effects of biochar and DMPP on NH_4_^+^ and NO_3_^−^ contents in soil under different moisture conditions. N, urea; C, biochar; DMPP, dimethyl-phenyl-piperazinium, a nitrification inhibitor. **(a)** soil NH_4_^+^-N concentration under 200% WHC; **(b)** soil NO_3_-N concentration under 200% WHC; **(a)** soil NH_4_^+^-N concentration under 60% WHC; **(b)** soil NO_3_-N concentration under 60% WHC. Error bars indicate standard deviation of replicates (*n* = 3).

Figure 1 shows the changes in NH_4_^+^-N concentration during the incubation period. Urea which applied to the soil decomposes under the action of soil urease. In general, the concentration of NH_4_^+^-N in the soil showed the similar trend. The peak value of NH_4_^+^-N in soil under 60% WHC occurred on the 7th day, which was 333.14 mg kg^−1^ under N + C + DMPP treatment, and the lowest NH_4_^+^-N concentration reached 268.30 mg kg^−1^ under N + C treatment except CK. Under 200% WHC, the NH_4_^+^-N concentration of all soil treatments peaked at the 14th day. The highest NH_4_^+^-N concentration appeared in N + C + DMPP treatment, reaching 286.71 mg kg^−1^, and the lowest appeared in N + C treatment, which was 95.57 mg kg^−1^. Compared with the N treatment, the N + C treatment decreased the NH_4_^+^-N content, and the application of DMPP significantly increased the NH_4_^+^-N concentration.

#### 3.1.2 NO_3_^−^-N content

The change in the NO_3_^−^-N concentration during the incubation period is shown in Figure 1. The figure shows that the concentration of NO_3_^−^-N increased with time. Under 60% WHC, the NO_3_^−^-N concentration treated with N and N + C increased rapidly from 7 to 21 days after adding the nitrogen source and then returned to stability, which was significantly higher than that under DMPP treatment. From day 14 to 21, the NO_3_^−^-N content of each DMPP treatment increased rapidly, The lowest NO_3_^−^-N concentration in the N + DMPP treatment (except CK) was 170.18 mg kg^−1^. From day 28 to 42, the NO_3_^−^-N concentration of each treatment remained in the range of 164.83 to 231.8 mg kg^−1^. Under 200% WHC, the NO_3_^−^-N content at 0–14 days in the N + C and N treatments increased rapidly, and the rate of increase with biochar addition was slightly lower. The increase rate of the DMPP treatment was relatively slow and stabilized in the range of 98.658–130.956 mg kg^−1^ after 35 days. The application of DMPP alone or in combination with biochar can effectively slow nitrification, and the inhibitory effect is more significant at 200% WHC.

The primary factors influencing soil nitrification encompass soil moisture, aeration conditions, the *amoA* gene abundance of AOA and AOB, soil pH, and the content of soil organic matter (Ren et al., 2023). In this study, we found that the N + C treatment could reduce the NH_4_^+^-N concentration in the soil under 60% WHC culture, increase the nitrification rate constant, indicating that the addition of straw biochar could improve the nitrification of the soil. Biochar effectively improved the soil aeration status, which was conducive to the accelerated growth and reproduction of AOB, and increased their activity, which was conducive to nitrification (Hu et al., 2024). Biochar could increase acid soil pH value and weak the decrease in soil pH, which can directly or indirectly affect the nitrification of soil (Aurangzeib et al., 2024).

### 3.2 Changes in AOA and AOB gene abundance of biochar and DMPP under different water content

**Figure 2:**
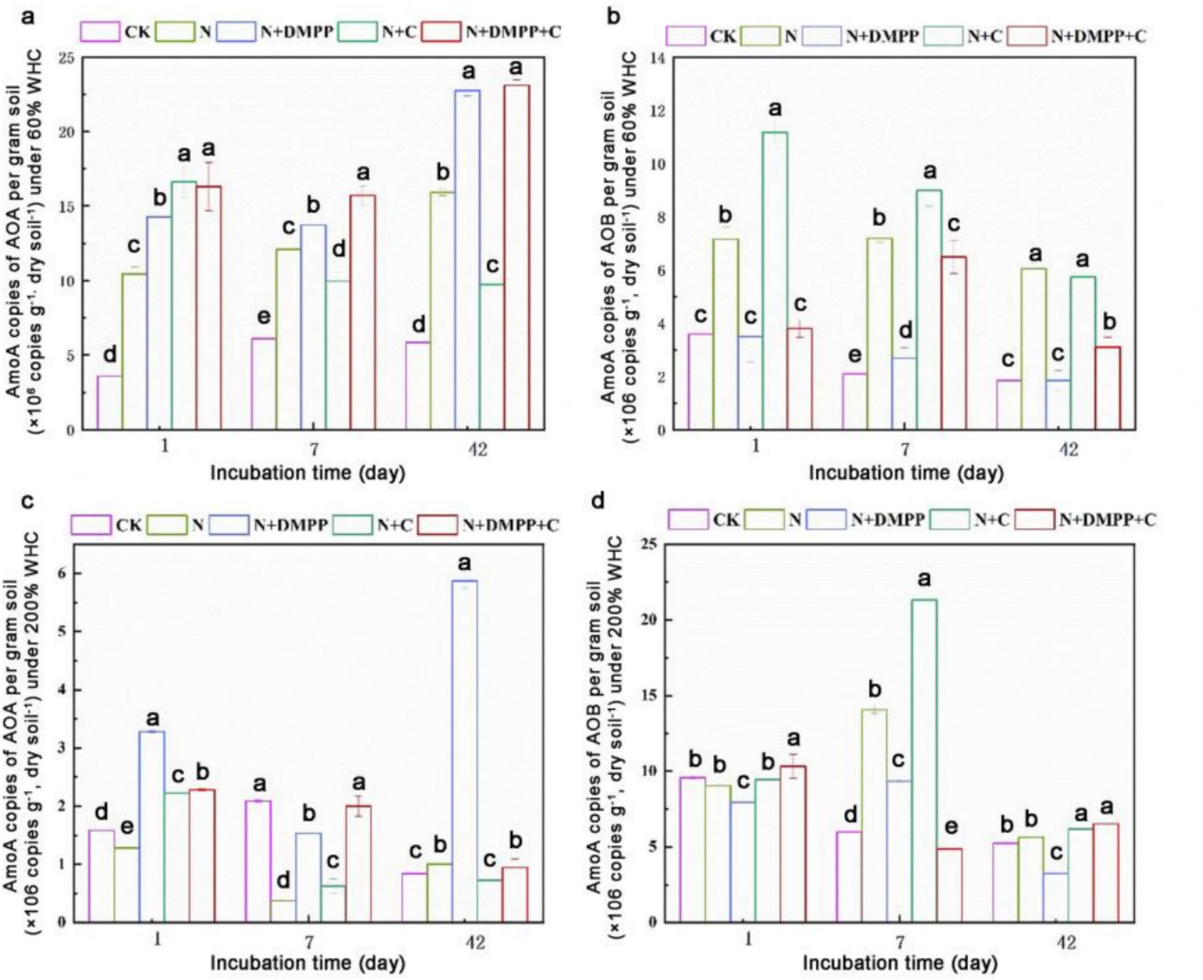
Effects of biochar and DMPP on AOA and AOB quantity in soil under different moisture conditions. N, urea; C, biochar; DMPP, dimethyl-phenyl-piperazinium, a nitrification inhibitor; AOB, ammonia-oxidizing bacteria; AOA, ammonia-oxidizing archaea. **(a)** AmoA copies of AOA per gram soil under 60% WHC; **(b)** AmoA copies of AOB per gram soil under 60% WHC; **(c)** AmoA copies of AOA per gram soil under 200% WHC; **(d)** AmoA copies of AOB per gram soil under 200% WHC; Error bars indicate standard deviation of replicates (*n* = 3). Letters above bars indicate a significant difference (*p* < 0.05) between treatments for each incubation day.

As shown in Figure 2, under 60% WHC conditions, there were significant differences in the copy number of *amoA* gene in AOB between the treatments. Compared to the N treatment, the copy number of *amoA* gene in AOB significantly increased in the N + C treatment on day 1 and 7. The N + DMPP and N + C + DMPP treatments had a significant inhibitory effect on the number of AOB, especially on day 1, when the inhibition rates were as high as 50.0 and 45.7%, respectively, compared with the N treatment. The number of AOB treated with N + DMPP on day 1 and 42 was not different from that in the CK treatment. The response of AOA to DMPP was opposite to that of AOB, and the amount of AOA under N + DMPP and N + C + DMPP treatments increased significantly during the entire culture period, especially on the 42 days.

Under 200% WHC conditions, compared with the N treatment, the N + C treatment increased *amoA* gene copy number of AOB by 51.53%, especially on the 7th day. N + DMPP inhibited the growth and reproduction of AOB. The response of soil AOA to nitrification inhibitors differs from that of AOB. During the entire incubation period, the growth and reproduction of AOA treated with DMPP were significantly promoted.

N and nitrification inhibitors play crucial roles in the soil ecosystem and have complex and significant impacts on the gene abundances AOA and AOB. It has been found that with the increase in the application rate of nitrogen fertilizers, the gene abundance of AOB shows an upward trend in the short term, which is because AOB has a strong affinity for higher-concentration NH_4_^+^-N, and can rapidly utilize these additional nitrogen sources for metabolism and proliferation (Ren et al., 2023). While, AOA often has stronger adaptability in acidic environments (Wu et al., 2020). DMPP can interfere with the key metabolic pathways of AOB, hinders their utilization of ammonium nitrogen, and thus affects their growth and reproduction. In contrast, due to the differences in metabolic pathways and ecological niches between AOA and AOB, AOA responds relatively weakly to DMPP (Liu et al., 2023). As a carbon-rich solid material, biochar, after entering the soil, exerts multi-dimensional impacts on the gene abundances of AOA and AOB by virtue of its unique physical and chemical properties. As a carbon-rich solid material, biochar, after entering the soil, exerts multi-dimensional impacts on the gene abundances of AOA and AOB by virtue of its unique physical and chemical properties. Biochar could improve soil aeration and WHC, which are conducive to the growth of aerobic AOB (Fang et al., 2021); while, AOA usually has certain advantages in acidic environments. Alkaline biochar may inhibit the growth of some AOA. Biochars prepared from different raw materials, as well as different soil types and environmental conditions, will all lead to differences in the influencing results, which still await further in-depth research.

### 3.3 Effects of biochar and DMPP on soil NH_3_ emission rate and cumulative emission under different moisture

**Figure 3:**
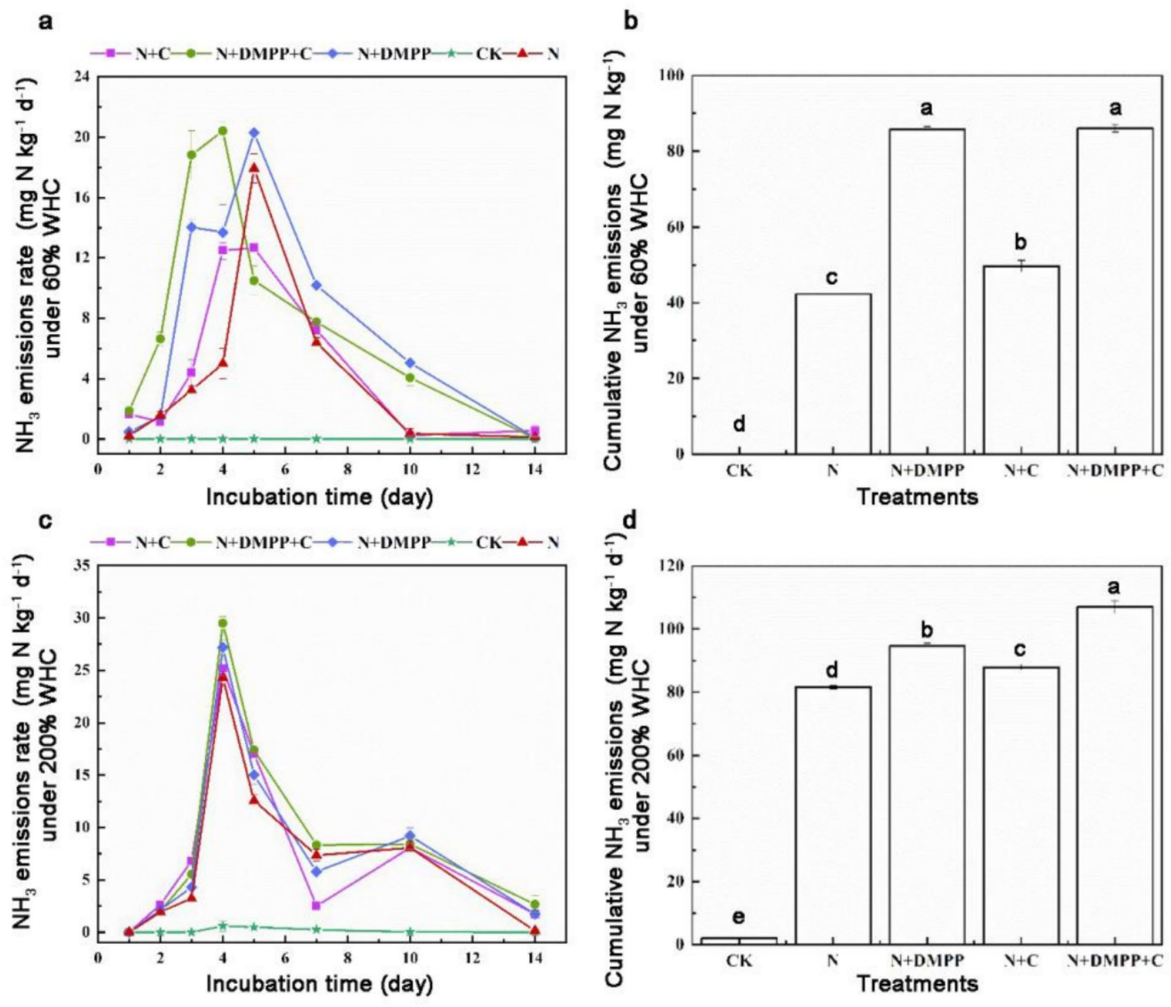
Effects of single or combined application of biochar and DMPP on NH_3_ emission rate and cumulative emission in soil under different moisture conditions. N, urea; C, biochar; DMPP, dimethyl-phenyl-piperazinium, a nitrification inhibitor. **(a)** NH_3_ emissions rate under 60% WHC; **(b)** cumulative NH_3_ emissions under 60% WHC; **(c)** NH_3_ emissions rate under 200% WHC; **(d)** cumulative NH_3_ emissions under 200% WHC. Error bars indicate standard deviation of replicates (*n* = 3). Letters above bars indicate a significant difference (*p* < 0.05) between treatments.

The NH_3_ emission rates under the two WHCS after the application of nitrogen sources are shown in Figure 3. The variation trend of the NH_3_ emission was essentially the same, showing an increase to the peak value and then a gradual decrease until no NH_3_ was detected.

Under 60% WHC water conditions, after adding N fertilizer for 3–5 days, the NH_3_ rate reached the peak, among which 17.93 mg N kg^−1^ in N treatment, and 12.67 mg N kg^−1^ in N + C treatment. The peak emission rates of N + DMPP and N + DMPP + C treatments were 20.3 and 20.41 mg N kg^−1^, respectively. Under 200% WHC water condition, all treatments reached the peak on the 4th day, among which the peak value was 24.3, 25.2, 27.17, 29.5 mg N kg^−1^ in treatment of N, N + C, N + DMPP, N + DMPP + C, respectively.

As can be seen from Figure 3, the addition of biochar, DMPP, or their combination had a significant effect on the cumulative loss of soil ammonia. Under 60% WHC water conditions, no ammonia was detected in the CK group (without N source added), NH_3_ loss was significantly increased under biochar and DMPP treatments. The maximum NH_3_ emissions appeared in the N + DMPP + C treatment which was 86 mg N kg^−1^ and N + DMPP treatment, which was 85.77 mg N kg^−1^. Compared with N treatment, NH_3_ loss in N + C was increased by17.51%, which was 49.67 mg N kg^−1^. When the water condition was 200% WHC, biochar and DMPP promoted the cumulative loss of ammonia under flooded conditions.

The above results show that the addition of biochar, a nitrification inhibitor, or their combination increased the cumulative ammonia volatilization in soil under the two moisture conditions, and the effect of DMPP on increasing ammonia volatilization was more significant under 60% WHC conditions. The cumulative volatilization of ammonia at 200% WHC was generally higher than that at 60% WHC, and the nitrification inhibitor DMPP alone tended to promote ammonia volatilization in different land-use types (He et al., 2018). The direct addition of biochar to soil inevitably affects the nitrogen cycle. The results of this study showed that biochar addition increased the cumulative NH_3_ volatilization under the two water conditions, which is consistent with the results of the study of Zhao et al. (2018), who found that total ammonia volatilization increased by approximately 102% after 10 seasons of biochar addition in a pot experiment of wheat/millet rotation. Previous studies have found that biochar has a large specific surface area and abundant oxygen-containing functional groups, which has a good adsorption effect on NH_3_ in dryland soil and can reduce the loss of soil ammonia volatilization (Zheng et al., 2018). The main reason that short-term biochar input significantly increased soil NH_3_ emissions may be that biochar can significantly increase soil pH and improve soil permeability (Castaldi et al., 2011).

DMPP increases ammonia volatilization which delays the nitrification reaction of NH_4_^+^-N, resulting in a higher concentration of NH_4_^+^-N in soil and surface water, thereby promoting ammonia loss (Liu et al., 2025). In this study, the cumulative volatilization of ammonia in the soil was increased by adding DMPP or combining with biochar under two WHCs condition, and the cumulative NH_3_ loss of the N + C + DMPP treatment was found to be higher than that of the biochar and DMPP treatments alone.

### 3.4 Effects of biochar and DMPP on soil N_2_O emission rate and cumulative emission under different moisture conditions

**Figure 4:**
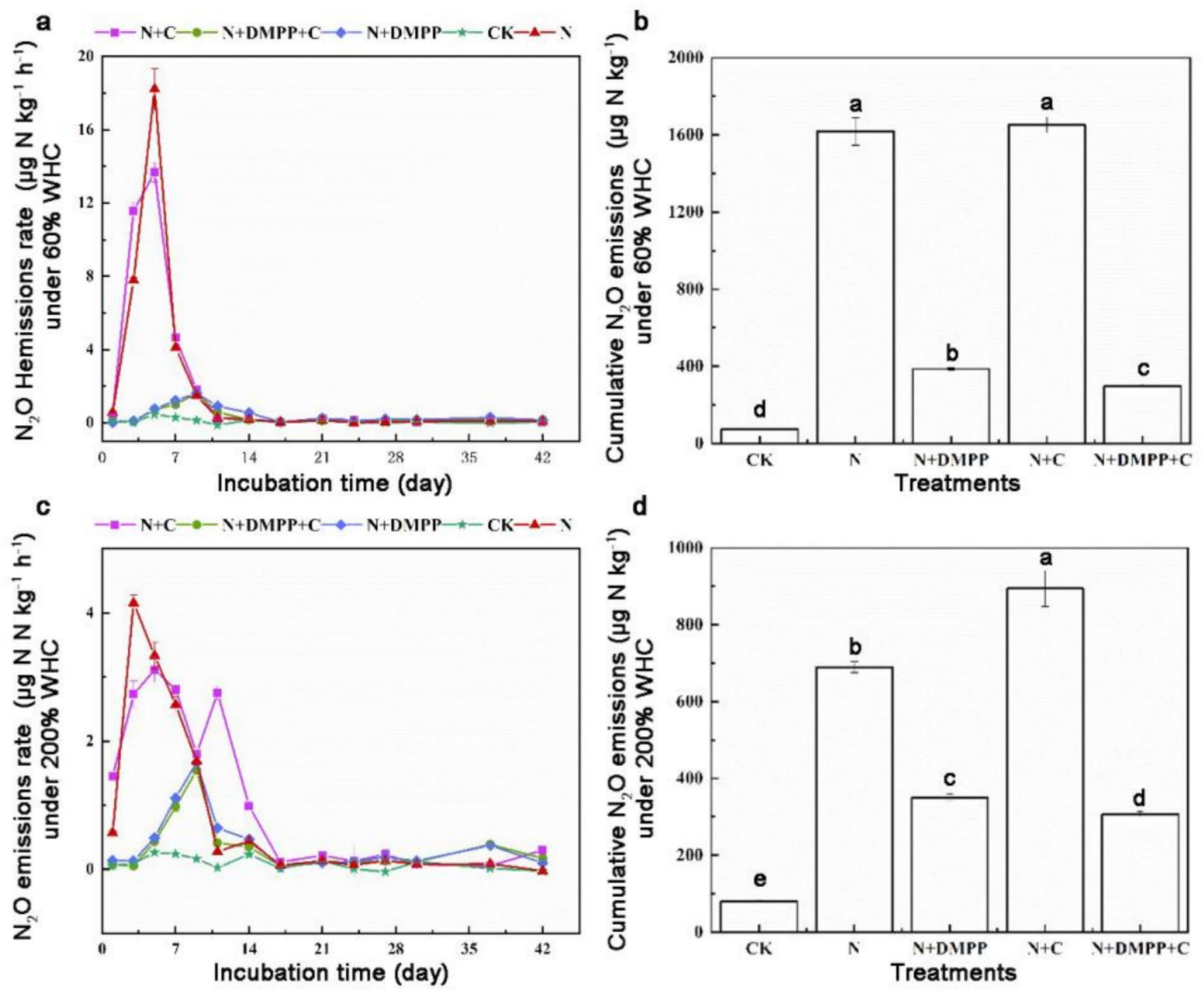
Effects of single or combined application of biochar and DMPP on N_2_O emission rate and cumulative emission in soil under different moisture conditions. N, urea; C, biochar; DMPP, dimethyl-phenyl-piperazinium, a nitrification inhibitor. **(a)** N_2_O emissions rate under 60% WHC; **(b)** cumulative N_2_O emissions under 60% WHC; **(c)** N_2_O emissions rate under 200% WHC; **(d)** cumulative N_2_O emissions under 200% WHC. Error bars indicate standard deviation of replicates (*n* = 3). Letters above bars indicate a significant difference (*p* < 0.05) between treatments.

As can be seen from Figure 4, under 60% WHC condition, peak N_2_O emission occurred in N + C treatment on the 5th day, which reached to 18.22 μg kg h^−1^. The emission peaks of N + DMPP + C and N + DMPP treatments appeared on the 9th day, only 1.70 and 1.60 μg kg h^−1^, respectively. DMPP, alone or in combination with biochar, can delay the peak time of N_2_O and reduce emission rate. Under 200% WHC condition, the cumulative emissions of N_2_O in all treatments ranged from 0.23 to 4.16 μg kg^−1^ (Figure 4). The peaks of N_2_O emissions in N + C, N + DMPP and N + DMPP + C treatments were 3.11, 1.67, and 1.54 μg kg h^−1^, respectively, which were reduced by 25.2, 59.9, and 63%, respectively, compared with that in N treatment.

Under 60% WHC, the cumulative emissions of N_2_O were 1617.27 μg kg^−1^ under N treatment and 1651.39 μg kg^−1^ under N + C treatment. DMPP alone and combination with BC significantly reduced cumulative emissions of N_2_O by 76.1 and 81.5% compared with N treatment, and the mixed application of biochar had a better effect on soil reduction. Under 200% WHC condition, N_2_O cumulative emissions under N treatment were 689.40 μg kg^−1^, and those were 893.9 μg kg^−1^ under N + C treatment, 350.16 μg kg^−1^ under N + DMPP treatment and 307.06 μg kg^−1^ under N + DMPP + C treatment, which were decreased by 49.2 and 55.5% when compared with N treatment, respectively.

The results showed that under 200% WHC, the N and N + C treatments decreased the accumulation of N_2_O by 57.4 and 45.9%, respectively, when compared with the 60% WHC treatment. An increase in water content could reduce the accumulation of N_2_O under conventional fertilization and biochar treatments. DMPP showed a better effect in reducing N_2_O emissions under both water conditions, and the effect of DMPP combined with biochar was better. As one of the most important greenhouse gases, N_2_O has a significant impact on global climate and environmental change. The N_2_O emission from farmland soils are generally produced through nitrification and denitrification by nitrifying microorganisms (Wu et al., 2024). In this experiment, the conversion process of NH_4_^+^ in soil under the 60% WHC conditions was carried out in aerobic culture, so the emission of N_2_O mainly came from the nitrification process of soil; under 200% WHC conditions, due to anaerobic waterlogging, denitrification was the main way to produce N_2_O under anaerobic or anoxic conditions. When the water content was low, biochar promoted the nitrification process of the soil, whereas when the water content was high, it led to the deterioration of soil ventilation and promoted denitrification. However, studies have also shown that when the water content is high, the anaerobic environment is conducive to the growth and reproduction of denitrifying microorganisms, promoting the denitrification of soil, increasing the emission of N_2_O. Alternatively, the higher ash content of biochar leads to an increase in soil N_2_O emissions.

The monitoring data in the wheat-maize rotation system in calcaric soil showed that the nitrification inhibitor DMPP can also effectively reduce soil N_2_O emissions, which is the same trend as the conclusion of this study (Zhao et al., 2017). Under the two water conditions, compared with the single application of DMPP, the cumulative emissions of N_2_O under the combined application of biochar and DMPP were further reduced to a significant level, indicating that DMPP could weaken the excitation effect of biochar addition on the nitrification process, thus further contributing to emission reduction. Some studies have also shown that the combined application of biochar and DMPP has N_2_O effect emissions in vegetable ecosystems, which may be related to the properties of the biochar, soil type, N_2_O, pH, moisture, texture, organic matter content, and other factors (Li et al., 2023).

### 3.5 Effects of biochar and DMPP on the greenhouse potential of N_2_O under different moisture conditions

**Figure 5:**
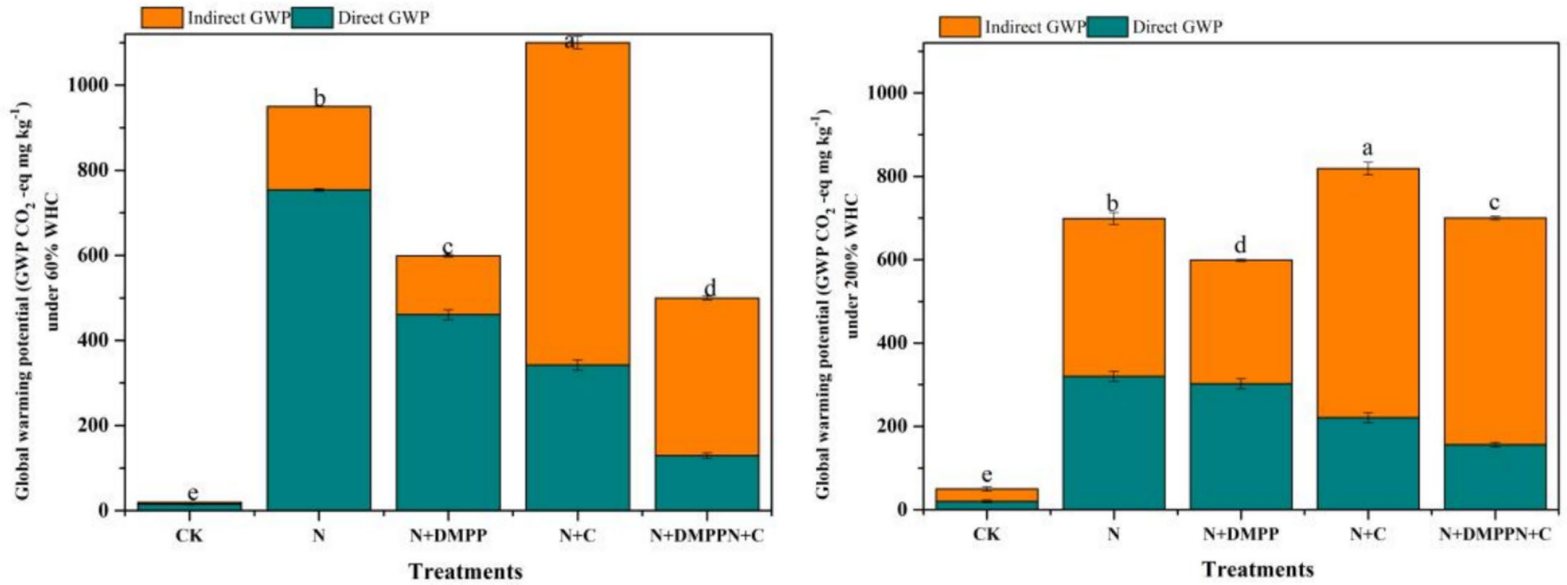
Effects of biochar and DMPP alone or in combination on global warming potentials (GWP). N, urea; C, biochar; DMPP, dimethyl-phenyl-piperazinium, a nitrification inhibitor. Error bars indicate standard deviation of replicates (*n* = 3). Letters above bars indicate a significant difference (*p* < 0.05) between treatments.

It can be seen from Figure 5 that under 60% WHC water condition, the greenhouse potential (GWP) of N + C treatment is 5.3% higher than that of N treatment. The GWP of the N + DMPP + C and N + DMPP treatments were significantly lower than that of the N treatment, with reduction rates of 43.2 and 39.0%, respectively. For the N, N + C, N + DMPP, and N + DMPP + C treatments, the contribution rates of direct N_2_O emissions to GWP were 79.3, 76.9, 31.1, and 25.8%, respectively, and the contribution rates of indirect N_2_O emissions (NH_3_ volatilization and resedimentation) to GWP were 20.7, 23.1, 70.0, and 74%, respectively. Direct N_2_O emissions from soil under the N and N + C treatments accounted for the main position of GWP, and indirect N_2_O emissions caused by NH_3_ volatilization in the two treatments with DMPP were the main greenhouse effect potential.

Under 200% WHC, the GWP of the N + C treatment was still the highest, 17.8% higher than that of the N treatment. Compared with the N treatment, the GWP of the N + DMPP + C and N + DMPP treatments were significantly reduced by 8.5 and 13.8%, respectively. For the N, N + C, N + DMPP, and N + DMPP + C treatments, the contribution rates of N_2_O direct emissions to the GWP were 45.8, 50.4, 27.0, and 22.3%, respectively, and the contribution rates of NH_3_ to the GWP were 54.3, 49.6, 73, and 77.7%, respectively. In conclusion, the indirect emission of N_2_O caused by NH_3_ volatilization in soil with nitrification inhibitors occupied a dominant position in the greenhouse gas warming potential of the soil. The GWP of the N + DMPP and N + C + DMPP treatments were 13.8 and 8.5% lower than that of N. Du et al. (2019) showed that the contribution ratio of N_2_O to the GWP was only 5.25% on average. In this study, the direct N_2_O emission of soil under N and N + C treatment was 76.9%–79.3% under 60% WHC, accounting for the main position of soil greenhouse gas warming potential. Approximately 1% (0.2–5%) of the discharged NH_3_-N is deposited on land and converted into N_2_O. When considering the indirect emission of N_2_O related to NH_3_ volatilization in the current study, the contribution rates of NH_3_ to GWP in the soils treated with N + DMPP and N + DMPP + C were 70.0 and 74%, respectively. The indirect emission of N_2_O caused by ammonia volatilization occupies a dominant position in soil GHG warming potential. Many studies have shown that biochar application can effectively reduce GWP (Yan et al., 2020) in the first year, which differs from the conclusions of this study. The main reason for this is that the N + C treatment in this experiment promoted both N_2_O emission and ammonia volatilization, resulting in increased emissions of active nitrogen gas, thus increasing the greenhouse effect potential of direct and indirect emissions of N_2_O. Under 200% WHC conditions, N_2_O emissions from GWP treated with N, N + C and N_2_O indirect emission caused by ammonia volatilization occupied the same position, and N_2_O indirect emission caused by N + DMPP and N + DMPP + C treated with ammonia volatilization still occupied the dominant position of soil GWP. However, this study did not consider the warming potentials of the CO_2_ and CH_4_ gases. Future studies should comprehensively consider the greenhouse effect potential of active nitrogen gases, CO_2_ and CH_4_, and the long-term combined application of biochar and inhibitors to reduce the warming potential of active nitrogen gases in farmland.

## 4 Conclusion

The addition of biochar promoted nitrification and ammonia volatilization in soil under both types of water, and the loss of ammonia volatilization was more significant under flooding conditions. Under both water conditions, DMPP alone significantly enhanced the mitigation of N_2_O emissions. While, the cumulative emissions of N_2_O were further reduced under the combined application of biochar and DMPP and reached a significant level. The addition of biochar alone increased the greenhouse potential of the soil under both 60 and 200% WHC conditions, while nitrification inhibitors alone or in combination with biochar can significantly reduce the greenhouse effect potential. To maximize N_2_O mitigation and reduce GWP, biochar and DMPP should be applied together, particularly in soils with moderate moisture levels (e.g., 60% WHC) and regular monitoring of AOB and AOA populations is recommended to assess the long-term effects of DMPP and biochar on soil nitrification processes and greenhouse gas emissions.

## Data Availability

The original contributions presented in the study are included in the article/supplementary material, further inquiries can be directed to the corresponding authors.
